# Sphingosine-mediated death of *Pseudomonas aeruginosa* involves degradation of cardiolipin by the maintenance of outer lipid asymmetry system

**DOI:** 10.1128/iai.00591-24

**Published:** 2025-03-10

**Authors:** Heike Grassmé, Gregory C. Wilson, Yuqing Wu, Mike Hasenberg, Simone Keitsch, Federico Caicci, Michael J. Edwards, Ildiko Szabo, Erich Gulbins

**Affiliations:** 1 Institute of Molecular Biology, University Hospital Essen, University of Duisburg-Essen 27170 , Duisburg, North Rhine-Westphalia, Germany; 2 Department of Surgery, University of Cincinnati College of Medicine 12303 , Cincinnati, Ohio, USA; 3 Imaging Center Essen (IMCES), Electron Microscopy Unit (EMU), University of Duisburg-Essen 27170 , Duisburg, North Rhine-Westphalia, Germany; 4 Department of Biology, University of Padova, Padova, Italy; Stanford University School of Medicine, Stanford, California, USA

**Keywords:** *Pseudomonas aeruginosa*, sphingosine, cardiolipin, lipase, membrane asymmetry, Mla system

## Abstract

Respiratory infections with multiresistant *Pseudomonas aeruginosa* are a major clinical problem, affecting mainly patients with pre-existing lung diseases such as cystic fibrosis (CF) or chronic obstructive pulmonary disease but also immunocompromised or elderly patients. We have previously shown that sphingosine, which is abundantly present on epithelial cells of the respiratory tract in healthy humans and wild-type mice, but almost undetectable on the surface of epithelial cells of the respiratory tract from CF patients and CF mice, efficiently kills many bacterial species *in vitro* and *in vivo*. Here, we show that sphingosine very rapidly induces marked changes in the membrane of *P. aeruginosa* with a rolling of the membrane followed by destruction of the bacteria. Sphingosine induced a degradation of cardiolipin via the maintenance of lipid asymmetry (Mla) system in *P. aeruginosa*. Degradation of cardiolipin induced by sphingosine is prevented in *P. aeruginosa* mutants of MlaY and reduced in mutants of MlaZ and MlaA. Mutants of MlaY and MlaZ were resistant to sphingosine-induced death of *P. aeruginosa*. In summary, our data indicate that sphingosine induces the death of *P. aeruginosa* by a persisting degradation of cardiolipin by the Mla system leading to severe membrane changes in bacteria, while leaving mammalian cells, devoid of cardiolipin in their plasma membrane, alive.

## INTRODUCTION

*Pseudomonas aeruginosa* are ubiquitous and opportunistic pathogens that cause severe respiratory tract and systemic infections, especially among patients with cystic fibrosis (CF), after previous viral infections, burn wounds, trauma, or sepsis, and in those requiring mechanical ventilation (1–5). Worldwide, *P. aeruginosa* has become the most common gram-negative pathogen associated with community-acquired pneumonia (17% of all cases), nosocomial pneumonia (25% of all cases) (6), and ventilator-associated pneumonia in intensive care units (28% of all cases) (7). Reported mortality rates associated with ventilator-associated pneumonia range from 33% to 72% (7). However, the most important are *P. aeruginosa* and *Staphylococcus aureus* infections among patients with CF. CF, which is caused by mutations of the cystic fibrosis transmembrane conductance regulator gene (human, *CFTR*; murine, *Cftr*), is the most common recessively inherited disorder in North America and Europe, with more than 80,000 CF patients in the EU and the USA alone (8). The most frequent cause of morbidity and mortality among CF patients is chronic pulmonary infection with bacterial pathogens, in particular *P. aeruginosa* and *S. aureus* (e.g., 9, 10), even after the introduction of drugs improving CFTR functions. In addition, many *P. aeruginosa* and *S. aureus* strains are highly resistant to existing antibiotics, and attempts to eliminate pulmonary *P. aeruginosa* or *S. aureus* among CF patients usually fail. Thus, it is important to develop novel strategies for treating pulmonary infections with *P. aeruginosa* and *S. aureus*.

We have previously demonstrated that sphingosine efficiently kills many bacterial species *in vitro* and *in vivo*, including *P. aeruginosa*, *Staphylococcus aureus* (even MRSA), *Acinetobacter baumannii*, *Haemophilus influenzae*, *Burkholderia cepacia*, *Moraxella catarrhalis,* and *Mycobacterium abscessus* (11, 12). Other groups have shown that sphingosine also kills *Escherichia coli*, *Neisseria meningitidis*, and *Porphyromonas gingivalis* (13–16). Our studies found that sphingosine is abundantly expressed on the luminal surface of nasal, tracheal, and bronchial epithelial cells in healthy human subjects or wild-type mice, whereas it is almost undetectable on the surface of epithelial cells of the respiratory tract from CF patients and CF mice (11, 12). Treating CF mice with inhaled sphingosine eliminated existing acute and chronic pulmonary *P. aeruginosa* infections and prevented new *P. aeruginosa* or *S. aureus* infections in these mice (11, 12, 17), a finding demonstrating that sphingosine plays a key role in the innate and immediate defense of the upper respiratory tract. Likewise, inhalation of recombinant human acid ceramidase by CF mice restored epithelial airway sphingosine levels and reversed acute and chronic infections with *P. aeruginosa* (12). Further studies demonstrated that inhalation of mice or mini pigs with even high doses of sphingosine twice daily over 14 days or application into isolated ventilated pig lungs did not result in any damage or inflammation in the trachea, bronchi, or lungs but was able to kill *P. aeruginosa* if lungs were infected (11, 12, 18–20).

The outer membrane of *P. aeruginosa* is an asymmetric lipid bilayer with glycerophospholipids such as cardiolipin in the inner leaflet, while the outer leaflet is mainly composed of lipopolysaccharide (LPS). The strong hydrophilic and hydrophobic interactions between LPS molecules result in mechanical strength and robustness of the outer membrane protecting the bacteria from external threats. In addition, the hydrophobicity of LPS provides a hydrophobic barrier for molecules, for instance, many antibiotics. One defense mechanism of gram-negative bacteria consists of an increased synthesis of LPS, resulting in increased hydrophobicity and a reinforced diffusion barrier. However, a large number of smaller molecules such as most nutrients are able to pass the outer membrane. It is therefore important to maintain the structure of the outer membrane for proper functioning of the bacteria. If glycerolipids from the inner membrane reach the outer membrane, for instance, upon a change of the LPS network, the mechanical and hydrophobicity properties are altered. Bacteria prevent these potentially dangerous alterations by an immediate removal of the mis-localized glycerophospholipids, which is mediated by the maintenance of lipid asymmetry (Mla) pathway (21). In *P. aeruginosa*, this pathway consists of MlaZ and MlaY as well as MlaA, MlaC, and the complex MlaBDEF (21). MlaY is a phospholipase and homologous to the well-characterized *E. coli* PldA (21, 22). This phospholipase is an integral outer membrane protein that serves to hydrolyze glycerophospholipids in the outer leaflet of the outer membrane into lysoglycerophospholipids (22). MlaZ is very likely a transporter protein that promotes the transport of glycerophospholipids from the outer leaflet of the outer membrane to MlaY, followed by degradation of the lipids (21). Since lysoglycerophospholipids exhibit detergent-like properties, they are immediately transported via the MlaA, MlaC, and MlaBDEF proteins to the inner cell membrane, where they are recycled (21). However, it is also possible that lipids are released into the periplasm upon cleavage by MlaY (21). MlaA functions as a channel for outer leaflet glycerophospholipids, MlaC is a transporter protein, and MlaBDEF serves to recycle the lipids (21). The MlaA, MlaC, and MlaBDEF pathways in *P. aeruginosa* might also serve to maintain the integrity of the outer leaflet of the outer membrane by transporting glycerophospholipids such as cardiolipin from the outer leaflet of the outer membrane to the inner membrane where they can be recycled. In summary, *P. aeruginosa* express two independent systems to remove glycerophospholipids such as cardiolipin from the outer leaflet of the outer membrane, i.e., (i) the MlaA, MlaC, and MlaBDEF system that transports glycerophospholipids from the outer leaflet of the outer membrane to the inner cell membrane for recycling and (ii) the MlaZ and MlaY system that degrades glycerophospholipids once they are mislocated to the outer leaflet of the outer membrane.

We have previously shown that treatment of several *P. aeruginosa* and *S. aureus* strains with sphingosine resulted in a very rapid, i.e., within minutes, permeabilization of the bacterial plasma membrane, leakiness of the bacterial cells, loss of ATP, and bacterial metabolic activity (23). Mechanistically, we demonstrated that the presence of the protonated NH_2_ group in sphingosine, which is an amino alcohol, is required for sphingosine’s bactericidal activity, while the hydroxy group does not seem to be necessary for triggering bacterial cell death (23). In direct binding studies using immobilized sphingosine or cardiolipin or artificial liposomes, respectively, we demonstrated that sphingosine binds via its protonated NH_2_ group to the highly negatively charged lipid cardiolipin in bacterial plasma membranes (23).

Here, we investigated whether sphingosine induced the destruction of *P. aeruginosa* membranes and the degradation of cardiolipin by targeting the bacterial Mla system. We demonstrate that the degradation of cardiolipin induced by sphingosine is prevented in *P. aeruginosa* mutants of MlaY and reduced in mutants of MlaZ and MlaA. Finally, we show that these mutants of MlaY, MlaA, and MlaZ are resistant to sphingosine-induced cell death.

## RESULTS

**Fig 1:**
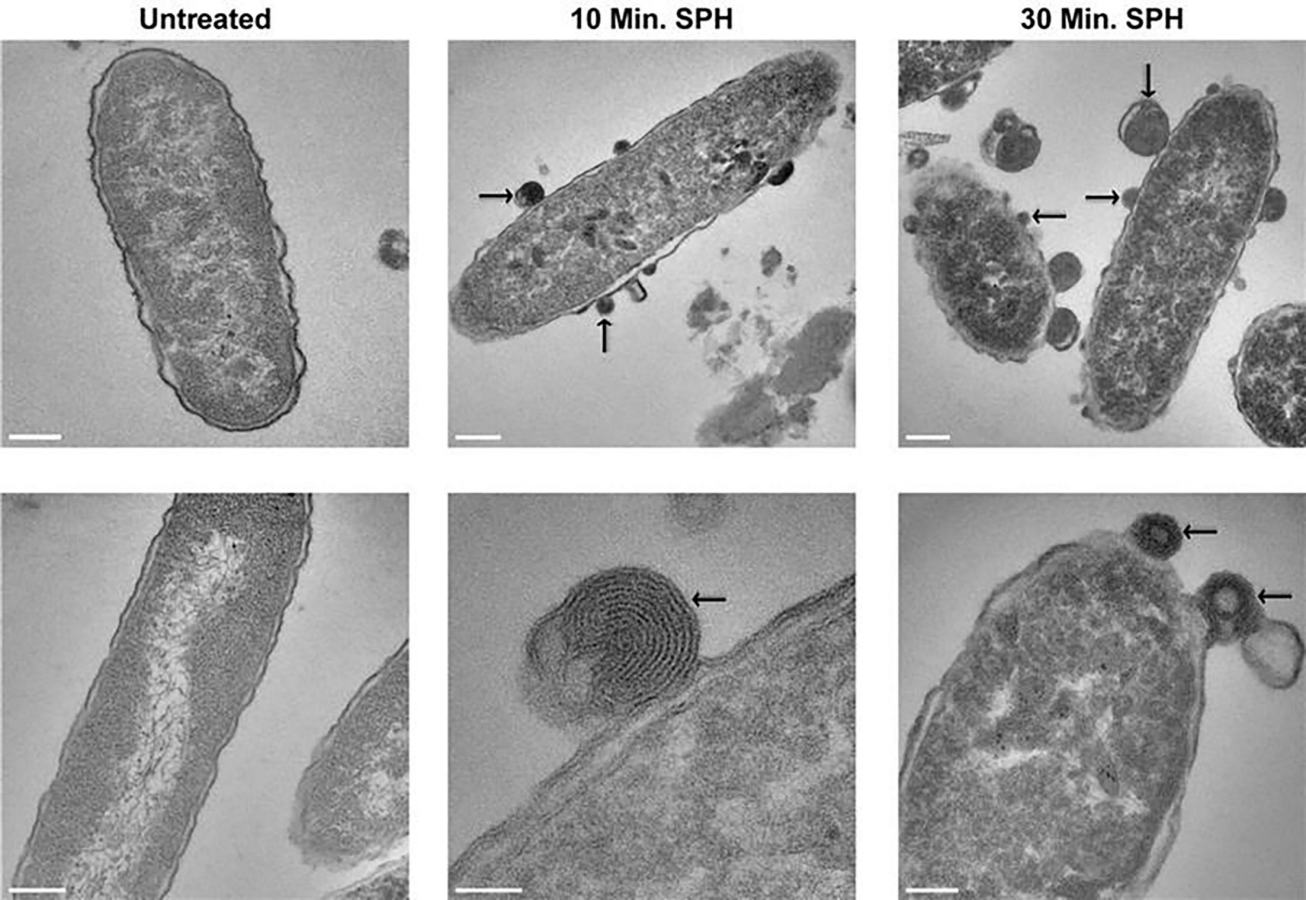
Electron microscopy of *P. aeruginosa* after treatment with sphingosine. *P. aeruginosa* ATCC 27853 were treated with 20 µM sphingosine (SPH) for 10 min or 30 min or left untreated, fixed, embedded, and analyzed by electron microscopy. A higher concentration of sphingosine was used because 10^8^ CFU *P*. *aeruginosa* were used in these experiments to obtain a pellet, which we were able to process. Shown is each a representative picture from at least 50 visualized cells. The arrows in the images in the upper row indicate some of the membrane blebs, and the arrows in the image in the lower row indicate typical membrane rolls. Scale bar sizes in the images: left column (both images) and middle and right column (upper images), each 200 nm; middle column (lower image), 50 nm; right column (lower image), 100 nm.

To identify molecular mechanisms that mediate sphingosine-induced killing of *P. aeruginosa*, we performed electron microscopy studies. We incubated the *P. aeruginosa* strain ATCC 27853 with sphingosine, fixed, embedded the bacteria, and visualized the bacterial structures in detail using transmission electron microscopy. These studies discovered remarkable changes in the bacterial membrane already 10 min after the addition of sphingosine. Sphingosine induced a rolling of the membrane and the formation of novel intracellular membranes (Fig. 1). The marked rolling very likely explains the permeabilization of the bacteria after treatment with sphingosine, while the increase of intracellular membranes might be an attempt of the bacteria to repair the membrane. At 30 min after treatment with sphingosine, we observed the death of the bacteria (Fig. 1). To get an impression of the three-dimensional bacterial morphology and ultrastructure, we also performed TEM tomography analyses from *P. aeruginosa* ATCC 27853 after treatment with sphingosine for 10 min (Movie S1). The results visualized the formation of membrane rolls that are finally released from the bacterium and the formation of newly formed intracellular membrane structures. These findings excluded that sphingosine simply induces a lysis of the cells.

**Fig 2:**
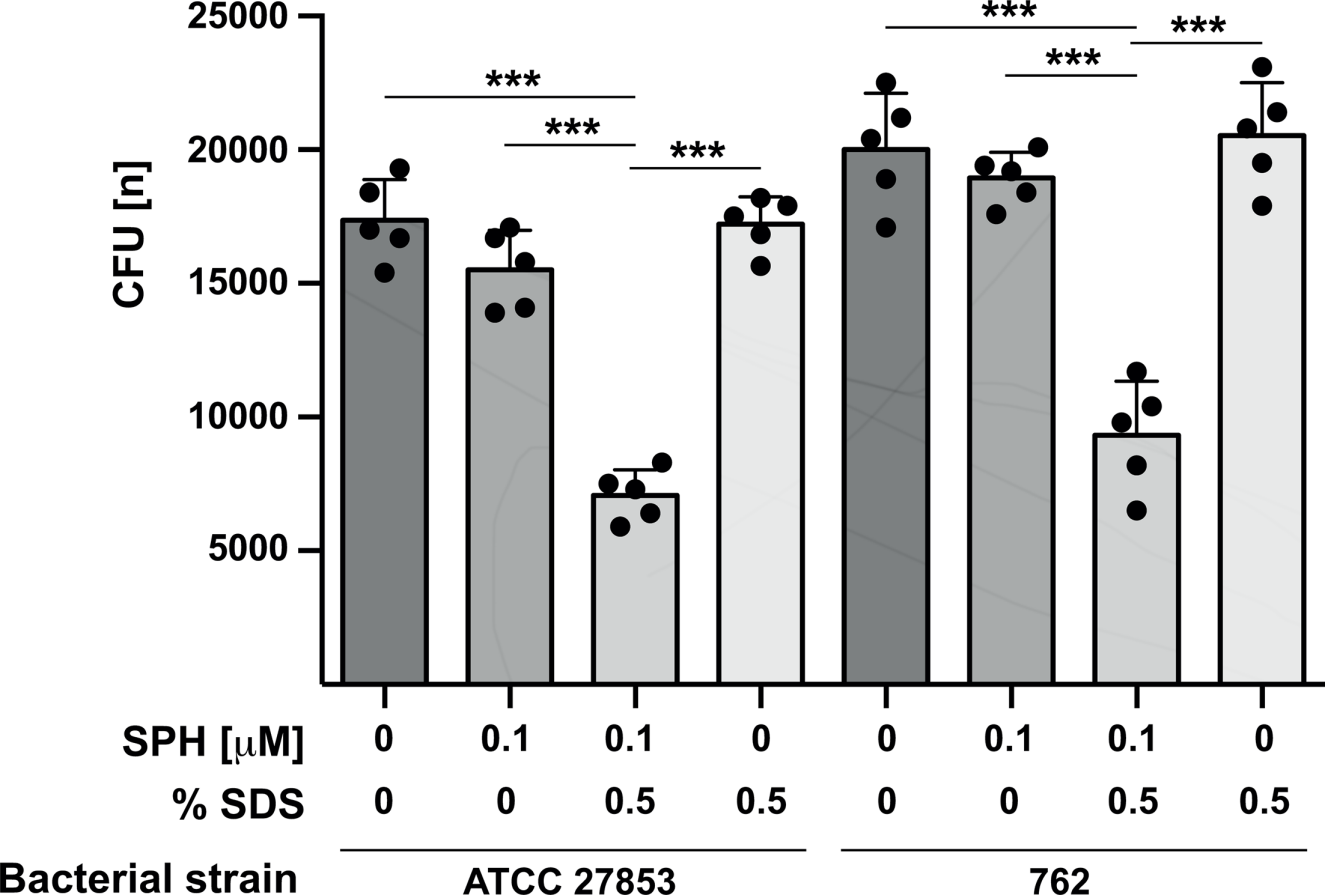
Sphingosine alters the permeability of the outer bacterial membrane. *P. aeruginosa* ATCC 27853 or 762 were incubated with 0.1 µM sphingosine or left untreated for 15 min in H/S followed by 2 h incubation in 10 mL TSB supplemented with 0.5% sodium dodecyl sulfate (SDS) and 0.5 mM EDTA. 0.5% SDS alone was without effect. Aliquots of the bacteria were plated on TSA, and CFUs were counted after o/n growth. Shown are the mean ± SD of five independent experiments; ^***^P <0.001, ANOVA.

These electron microscopy data suggest that sphingosine targets the bacterial membrane. To test whether sphingosine targets the outer bacterial membrane, we investigated whether sphingosine alters the permeability of the outer bacterial membrane. To this end, we incubated the bacteria with a very low dose of sphingosine (0.1 µM), which was too low to induce bacterial death by itself. We then treated the bacteria with a low dose of sodium dodecyl sulfate (SDS) (0.5% SDS), which lyses the bacteria, if the outer bacterial membrane is leaky and impaired, while it does not affect intact bacteria. The results revealed that pretreatment with sphingosine increased the permeability of the outer membrane for SDS and allowed 0.5% SDS to kill *P. aeruginosa* strains ATCC 27853, a laboratory strain, and 762, a clinical isolate, while 0.5% SDS alone was insufficient to kill *P. aeruginosa* (Fig. 2).

**Fig 3:**
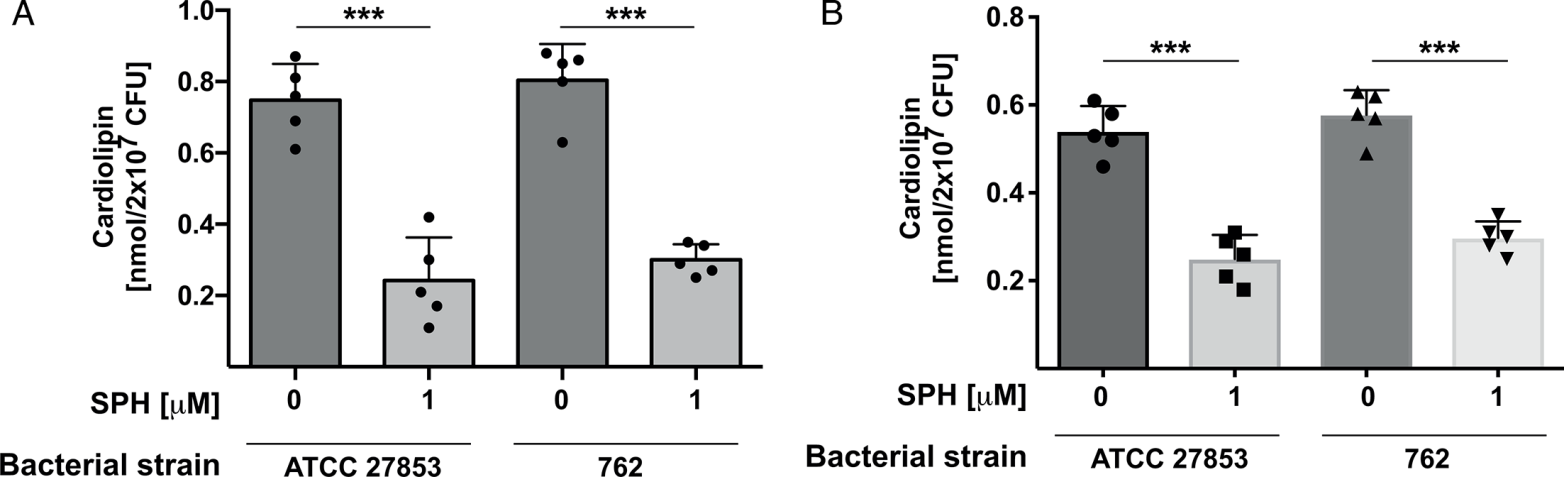
Sphingosine induces a degradation of cardiolipin. (**A**) *P. aeruginosa* strains ATCC 27853 or 762 (1 × 10^7^ CFU) were incubated in HEPES/saline (H/S, 132 mM NaCl, 20 mM HEPES [pH 7.0], 5 mM KCl, 1 mM CaCl_2_, 0.7 mM MgCl_2_, 0.8 mM MgSO_4_) for 20 min with 1 µM sphingosine (SPH) or left untreated, centrifuged, and lysed, and the cardiolipin content was determined by an enzyme kit from Abcam (#241036). Shown are the mean ± SD of five independent experiments; ^***^P <0.001, ANOVA. (**B**) *P. aeruginosa* strains ATCC 27853 or 762 (1 × 10^7^ CFU) were labeled with 40 µCi [^14^C]acetate/100 mL TSB, washed, treated for 20 min with 1 µM sphingosine (SPH) or left untreated, pelleted, and extracted, and lipids were analyzed by TLC. Displayed are the mean ± SD, five independent experiments; ^***^P <0.001, ANOVA, post hoc Tukey test.

These data suggest that sphingosine targets the outer bacterial membrane, and we therefore tested the following hypothesis: the positively charged NH_2_ group of sphingosine may bind to and displace cardiolipin from the inner to the outer leaflet of the outer bacterial membrane bringing it into close vicinity to the Mla system, in particular the phospholipase MlaY (21, 22). To verify this, we investigated whether sphingosine induces a decrease of cardiolipin in *P. aeruginosa*. To this end, we incubated 2 × 10^7^ colony-forming units (CFU) of the *P. aeruginosa* strains ATCC 27853 or 762 for 10 min with a low dose of sphingosine, i.e., 1 µM sphingosine, and then quantified the remaining cardiolipin in the bacteria using an enzymatic method to determine cardiolipin. The results show that sphingosine induced a massive, rapid decrease of cardiolipin in the bacteria (Fig. 3A). To confirm the consumption of cardiolipin, we labeled bacterial lipids by incubation of the bacteria with 40 µCi [^14^C]acetate [ARC 0173, 58 mCi/mmol]/100 mL tryptic soy broth (TSB) for 60 min, treated with sphingosine for 20 min or left the bacteria untreated, extracted, and separated lipids via thin-layer chromatography (TLC). The results confirm that sphingosine induced a rapid degradation of cardiolipin (Fig. 3B).

**Fig 4:**
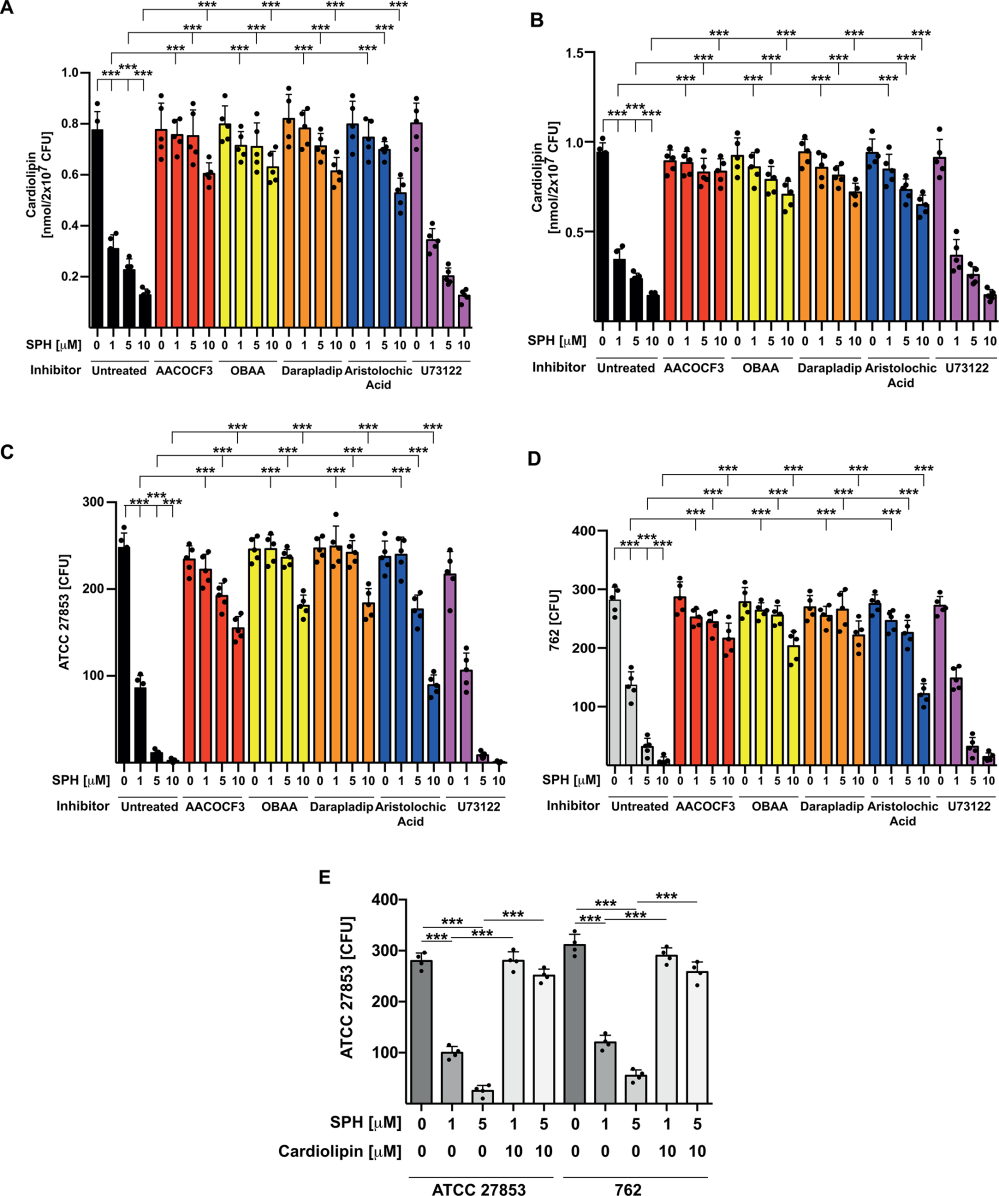
Inhibition of *P. aeruginosa* phospholipase A_2_ activity prevents sphingosine-induced death of the bacteria. (**A, B**) *P. aeruginosa* strains ATCC 27853 (**A**) and 762 (**B**) were grown to the early log phase and washed; 2 × 10^7^ CFU/sample was labeled with 40 µCi [^14^C]acetate/100 mL TSB, washed, and incubated with the PLA_2_ inhibitors AACOCF3 (25 µM), VU0364739 (25 µM), OBAA (25 µM), FIPI (50 µM), darapladip (50 µM), or aristolochic acid (5 µM) or the PLC inhibitor U73122 (10 µM), which served as negative control, or the samples were left untreated for 10 min. We then added sphingosine at 1, 5, or 10 µM or left the samples untreated. Bacteria were incubated for 15 min, and cardiolipin was determined by TLC after lipid extraction. Given are the mean ± SD of five independent experiments; ^***^P <0.001, ANOVA, post hoc Tukey test. (**C, D**) *P. aeruginosa* strains ATCC27853 (**C**) and 762 (**D**) were grown to the early log phase and washed, and 10,000 CFU/sample were incubated with the inhibitors as above or left untreated for 10 min. We then added sphingosine at 1, 5, or 10 µM or left the samples untreated. Bacteria were incubated for 45 min, aliquots were spotted on TSA plates, grown o/n, and the CFU were counted. Shown are the mean ± SD of five independent experiments; ^***^P <0.001, ANOVA, post hoc Tukey test. (**E**) Complementation of cardiolipin in ATCC 27853 or 762 *P*. *aeruginosa* reduced the bactericidal effects of 1 or 5 µM sphingosine. Bacteria were treated for 30 min with 10 µM cardiolipin prior to a 30 min treatment with 1 or 5 µM sphingosine in H/S, pH 7.0. Aliquots were then plated, and CFU were counted grown after o/n growth. Shown are the mean ± SD of four independent experiments; ^***^P <0.001, ANOVA, post hoc Tukey test.

Next, we tested the significance of the consumption of bacterial lipids by the outer membrane phospholipase (MlaY), which exhibits a phospholipase A_2_ (PLA_2_)-like activity. To this end, we incubated *P. aeruginosa* strains ATCC 27853 or 762 with a panel of different inhibitors of PLA_2_, i.e., AACOCF3, VU0364739, OBAA, FIPI, darapladip, or aristolochic acid, or left them untreated. If sphingosine induces cardiolipin degradation by activation of a phospholipase activity, these inhibitors may prevent the effects of sphingosine on cardiolipin degradation. The results show that all inhibitors of PLA_2_ reduced or abolished the effects of a 15 min treatment with the indicated concentrations of sphingosine on cardiolipin degradation in *P. aeruginosa* strains ATCC 27853 (Fig. 4A) or 762 (Fig. 4B).

Next, we tested whether inhibition of a PLA_2_ activity in *P. aeruginosa* strains ATCC 27853 and 762 also prevents sphingosine-induced cell death. Bacteria were treated with the inhibitors as above for 10 min or left them untreated, followed by incubation with 0, 1, 5, or 10 µM sphingosine for 45 min. Bacteria were washed and plated on TSA plates, and CFUs were counted after o/n growth. The results indicate that the PLA_2_ inhibitors prevented or reduced sphingosine-induced killing of *P. aeruginosa* ATCC 27853 (Fig. 4C) or 762 (Fig. 4D). The PLC inhibitor U73122 served as control and did not inhibit cardiolipin degradation or bacterial death upon sphingosine treatment (Fig. 4A through D).

The bactericidal effects of 1 or 5 µM sphingosine were also reduced by complementation of cardiolipin in ATCC 27853 or 762 *P*. *aeruginosa* upon incubation with 10 µM added cardiolipin prior to a 30 min treatment with 1 or 5 µM sphingosine (Fig. 4E).

**Fig 5:**
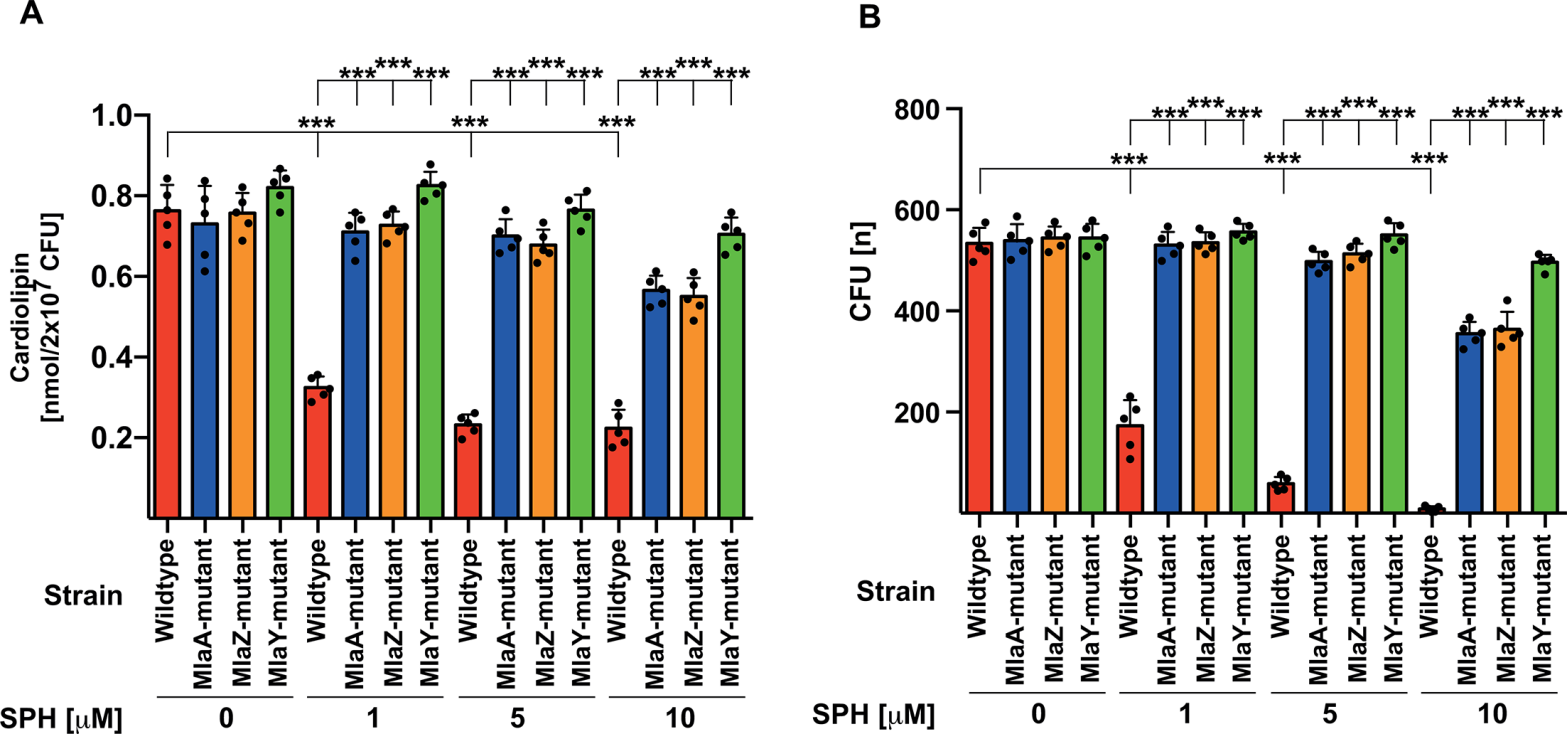
Mutants of lipid transport proteins MlaA and MlaZ or the lipase MlaY fail to degrade cardiolipin upon treatment with sphingosine and are relatively resistant to sphingosine. (**A**) To determine cardiolipin, the *P. aeruginosa* MlaA and MlaZ mutants, which are mutants of the lipid transport machinery for phospholipids, and the MlaY mutant, which functions as a lipase, and the corresponding wild-type strain were incubated with 1, 5, or 10 µM sphingosine for 15 min or left untreated; the bacteria were pelleted and extracted; and cardiolipin was determined as above. Mean ± SD, *n* = 5, ^***^P <0.001, ANOVA, and post hoc Tukey test. (**B**) 10,000 CFU of the above-described bacterial *P. aeruginosa* strains were incubated with 1, 5, or 10 µM sphingosine for 45 min or left untreated, and CFU were counted after o/n culture on agar plates. Given are the mean ± SD of five independent experiments; ^***^P <0.001, ANOVA, post hoc Tukey test.

To genetically prove that a specific phospholipase is important for sphingosine-mediated killing of *P. aeruginosa*, we employed mutants of the Mla pathway in *P. aeruginosa*. MlaA and MlaZ are transporters and serve to transport phospholipids between and through lipid bilayers, while MlaY functions as a lipase that serves to degrade lipids (21, 22). For this purpose, we quantified cardiolipin in the different bacterial strains and found that *P. aeruginosa* deficient for these proteins are relatively resistant to 1, 5, and 10 µM sphingosine (Fig. 5A). In accordance, cardiolipin degradation after treatment with sphingosine was greatly reduced in mutants for MlaA, MlaZ, and MlaY compared to the wild-type strain (Fig. 5A), which correlated with a reduced induction of death in the mutants compared to the wild-type bacteria (Fig. 5B). Resistance was highest in the lipase MlaY-deficient strain.

## DISCUSSION

In the present study, we show that incubation of *P. aeruginosa* with sphingosine induces a degradation of cardiolipin, which is mediated by a bacterial phospholipase, mainly MlaY (21, 22). The degradation of cardiolipin results in the death of the bacteria as shown in the experiments with PLA_2_ blockers and, more importantly, using genetic mutants of MlaY and the transport mutants MlaA and MlaZ. The results show that PLA_2_ inhibitors and the genetic deficiency of MlaY did not only block sphingosine-induced cardiolipin degradation but also the induction of bacterial death.

The Mla system usually serves to maintain the lipid asymmetry of bacterial membranes by the transport and degradation or reutilization of mis-localized lipids (21, 22). However, in the presence of sphingosine, this repair mechanism seems to be persistently activated and thereby results in a continuous degradation of cardiolipin with the final death of the bacteria.

Our data using low doses of SDS as a probe for the permeability of the outer bacterial membrane suggest that sphingosine increases the permeability of the outer bacterial membrane allowing access of SDS to the inner bacterial membrane and lysis of the bacteria (24). Collectively, these data indicate that sphingosine binds to cardiolipin, as previously shown (23), and induces a displacement of cardiolipin from the inner leaflet to the outer leaflet of the outer bacterial membrane, followed by the degradation of cardiolipin by MlaY. Sphingosine has been previously shown to bind to cardiolipin via its head group and to interact with the membrane via its acyl chain (24). This mode of interaction may induce a destabilization of the membrane and promote the transfer of cardiolipin complexed with sphingosine to the outer leaflet of the outer membrane.

However, our findings do not exclude that sphingosine also binds to other lipids, in addition to cardiolipin, and thereby mediates an increase in the permeability of the outer bacterial membrane. It might be possible that sphingosine binds to lipopolysaccharides and that this interaction promotes binding of sphingosine to cardiolipin, the translocation of cardiolipin to the outer leaflet, and its degradation.

In addition, binding of sphingosine to cardiolipin may solubilize and extract cardiolipin from the membrane resulting in direct degradation of MlaY in the intermembrane space.

Finally, it needs to be investigated in future studies whether sphingosine binds directly to MlaY, MlaA, and/or MlaZ and regulates the activity of these proteins.

Although our data show that sphingosine induces degradation of cardiolipin, the data do not fully clarify how the bacteria die. The electron microscopy studies indicate a rolling of the bacterial membrane and the formation of novel membranes within the bacteria upon incubation of the bacteria with sphingosine. Thus, the rolling of the membrane might be an attempt to remove sphingosine from the membrane, similar to the exclusion of permeability-altering toxins from biological membranes (25). However, due to the persistent effect of sphingosine, this repair overshoots and fails, and the bacteria die. The formation of intracellular membranes would be seen as an attempt by the bacteria to repair the inner and outer membrane.

In addition, the continuous consumption of cardiolipin may also result in a destabilization of the membrane resulting in the rolling of the membrane as observed in the EM studies: the positively charged sphingosine head group may bind to negatively charged molecules, as previously shown (23), in the outer leaflet of the outer and inner membrane, for instance, LPS or cardiolipin, while the acyl chains of the sphingosine molecules will form micelles. Since the headgroup of sphingosine is rather small (essentially a protonated NH_2_ group) and the negatively charged lipids are rather bulky, the formation of sphingosine micelles, bound to the surface or the outer and inner membrane, may result in a rolling of the membrane, which may only occur, if the membrane has been destabilized by prior consumption of cardiolipin.

Our observations also support the notion that sphingosine seems to act by a stoichiometric mechanism: if the concentration of sphingosine per membrane is too low, the bacteria are able to roll the affected membrane part containing complexes of sphingosine, for instance, with cardiolipin, release it, and repair the membrane, which then results in neutralization of sphingosine remaining in the released membrane vesicle. Thus, a critical concentration of sphingosine must be reached per bacterium to kill the bacteria.

The data showing a degradation of cardiolipin by sphingosine suggest a specific mode of action for sphingosine and do not support a simple detergent-like effect of sphingosine on the bacteria, at least at lower concentrations of sphingosine. However, at higher concentrations, sphingosine may also extract lipids, in particular cardiolipin from the bacterial membrane, and thereby kill *P. aeruginosa* independent of cardiolipin degradation.

While cardiolipin is present in the outer bacterial membrane, it is absent in the plasma membrane of mammalian cells, and only mitochondrial membranes contain cardiolipin (26). The absence of cardiolipin from mammalian plasma membranes may in part explain the finding that mammalian cells are rather resistant to sphingosine, at least cells of the respiratory tract, as we have previously shown (11, 12, 18–20, 27). In these studies, we inhaled high doses, i.e., up to 500 µM sphingosine, in mice or pigs or isolated perfused and ventilated pig lungs and did not observe any toxicity (11, 12, 18–20, 27). Exogenous sphingosine may also reach mammalian mitochondria, but mitochondria contain relatively high levels of sphingosine kinases (28, 29), which are not expressed in *P. aeruginosa*, and the very rapid phosphorylation of sphingosine within organelles of mammalian cells (29) might detoxify sphingosine and protect the cells from death.

These properties of sphingosine suggest that sphingosine is able to rapidly kill pathogens, for instance, in the respiratory tract without inducing an inflammation in the host/mammalian epithelial cells. This notion is consistent with the observation that high levels of sphingosine are detected in the plasma membranes of epithelial cells in the respiratory tract in healthy mice and humans, while sphingosine is reduced in the trachea and bronchi from mice or humans with cystic fibrosis. Inhalation of cystic fibrosis mice with sphingosine restores the ability of these mice to immediately kill *P. aeruginosa*, while untreated cystic fibrosis mice are highly sensitive to *P. aeruginosa* infections.

In addition to the bactericidal effect of sphingosine, sphingosine is also sensed by *P. aeruginosa*, mainly through a transcription factor, SphR, leading to expression of a gene with unknown function, SphA (30). Deletion of SphR increases sensitivity of *P. aeruginosa* to the bactericidal effects of sphingosine (30). Sphingosine might be also able to induce, via SphR, expression of PlcH, a secreted phospholipase C/sphingomyelinase, which is important for the virulence of *P. aeruginosa* (31). This response might be seen as a counter-attack of the bacteria to host-released sphingosine and might be in particular important in the defense of the bacteria to lower concentrations of sphingosine.

Very recent studies indicated that the *sphBCD* operon of *P. aeruginosa* might play an important role in the metabolism of sphingosine by *P. aeruginosa*, since its deletion reduced growth in the presence of sphingosine (32).

Collectively, these studies confirm our notion that sphingosine is able to kill *P. aeruginosa*. However, they also demonstrate additional sphingosine response elements in the bacteria. The specific response of the bacteria to sphingosine is very likely dependent on different application ways of sphingosine, for instance, as a micelle or attached to the surface of the incubation tube, the medium, or buffer, respectively, used during the treatment and certainly also the expression of genes in different strains.

An inhibition of MlaY by PLA2 inhibitors has not been previously published to the best of our knowledge. However, aristolochic acid and PACOCF3 have been shown to bind to and inhibit *E. coli* protein OMPLA (33), which is homologous to the *P. aeruginosa* MlaY protein (21). Several other observations support an action of PLA_2_ inhibitors on MlaY: studies by R.L. Guest et al. (21) indicated that an *E. coli* mutant that lacks *pldA* is sensitive to SDS/EDTA, which can be rescued either by re-expression of PldA or by expression of MlaY (together with MlaZ). Sequence analysis of MlaY indicates the presence of an α/β hydrolase domain (21), which is also present in PLA_2_ and targeted by, for instance, aristolochic acid (34, 35). The active site in the *E. coli* OMPLA contains Ser 144, His142, and Asn156 (33) and is similar to the active site in MlaY containing Ser 187, His 415, and Asp 388 (21). These observations support the notion that PLA_2_ inhibitors directly inhibit MlaY, but further structural studies are required to fully address this issue.

In summary, we show that sphingosine induces a degradation of cardiolipin in *P. aeruginosa*. Genetic and pharmacological data indicate that the lipase MlaY mediates degradation of cardiolipin in *P. aeruginosa* upon incubation with sphingosine. The genetic data also indicate an involvement of the lipid transporter MlaA and MlaZ proteins. The studies further show that genetic mutants of MlaY, A, and Z are resistant to sphingosine-induced death, which is characterized by marked changes of the bacterial membrane.

## MATERIALS AND METHODS

### Strains

The MlaA, MlaZ, and MlaY mutants of PAO-1 as well as the wild-type control strain PAO-1 were obtained from Dr. Salipante, U. of Washington, Seattle, USA (36). Gene names were PA2800, PA3238, and PA3239 (36). We did not observe any defects of growth of the mutants under optimal conditions, for instance, in TSB or LB medium, which is consistent with a previous publication (21).

In addition, we used the laboratory strain ATCC 27853 and the clinical isolate 762, which was isolated from a patient with *P. aeruginosa* sepsis (37).

### Sphingosine preparation

Sphingosine (Avanti Polar Lipids, #860490P) was suspended at 1 mM in 0.9% NaCl and sonicated using a tip sonicator (QSonica) to obtain a stable suspension. The stocks were frozen at −20°C. Before use, we sonicated the sphingosine solution again for 10 min in a bath sonicator (Bandelin, Sonorex) and then diluted it in HEPES/saline (H/S, 132 mM NaCl, 20 mM HEPES [pH 7.0], 5 mM KCl, 1 mM CaCl_2_, 0.7 mM MgCl_2_, 0.8 mM MgSO_4_) to reach the intended concentrations of 1 µM, 5 µM, or 10 µM sphingosine.

### Electron microscopy

10^8^ CFU *P*. *aeruginosa* ATCC 27853 were treated with sphingosine (20 µM) for 10 or 30 min, washed, fixed with 2.5% glutaraldehyde in 0.1 M sodium cacodylate buffer, pH 7.4 o/n at 4°C, and then postfixed with 1% osmium tetroxide plus potassium ferrocyanide 1% in 0.1 M sodium cacodylate buffer for 1 h at 4°C. After three washes, samples were dehydrated in a graded ethanol series and embedded in an epoxy resin (Epoxy Embedding Medium kit, Sigma-Aldrich).

Ultrathin sections with a diameter of 60 nm were cut using an ultramicrotome (EM UC7, Leica Microsystems, Wetzlar, Germany) equipped with a diamond knife (ultra 35°, Diatome, Nidau, Switzerland). Sections were collected on 200 mesh hexagonal thin-bar TEM copper grids (Plano GmbH, Wetzlar, Germany) and post-contrasted with 4% aqueous uranyl acetate and Reynold’s lead citrate solution using an automated contrasting instrument (EM AC20, Leica Microsystems, Wetzlar, Germany). Image acquisition was performed with a JEM 1400Plus TEM (JEOL Ltd., Tokyo, Japan), operating at 120 kV and equipped with a 4096 × 4096 pixels CMOS camera (TemCam-F416, TVIPS, Gauting, Germany) using SerialEM (v3.8.0) (38). The resulting 16-bit TIFF images were then processed in Fiji (v1.54f) (39). First, all images were converted to 8-bit, and image contrast was enhanced by normalization with 0.1% saturated pixels. Next, the images were smoothed using a median filter with a radius of 2 px, before a pseudo-flat-field correction was carried out, using the “Calculator Plus” function of the software. For this, a pseudo-background image was created by blurring an image copy with a Gaussian blur filter (Sigma (Radius) = 800). The resulting flat-field-corrected image was again smoothed (Gaussian blur filter (Sigma (Radius) = 2), and a scale bar was imprinted using the Fiji scale bar tool.

TEM tomography was carried out on 200 nm thick sections cut from the blocks prepared for ultrathin sectioning as described above. Sections were mounted on Formavar®-coated 2 × 1 mm slot copper grids (Plano, Wetzlar, Germany) and were counterstained with uranyl acetate. Right before image acquisition, a small drop of 6 nm nanogold particles suspended in deionized water was applied onto the section surface to serve as fiducial markers during reconstruction. As soon as the suspension drop was air-dried, the samples were examined with the JEOL JEM 1400Plus TEM, operating at 120 kV. Tilt series were acquired automatically over an angular range of −60° to +60° at 2° increments using SerialEM. Stack alignment and reconstruction by filtered back-projection were carried out using the software package iMOD (Ver. 4.9.7) (40).

### Growth and treatment of bacteria

All bacteria were stored as frozen glycerin stocks. Small aliquots from the frozen stocks were plated on tryptic soy broth agar plates, and the plates were grown overnight at 37°C. To grow the bacteria to the early logarithmic phase allowing reproducible growth conditions, we removed bacteria from the agar plate, suspended them in 40 mL TSB, and adjusted the optical density (OD_550_) of bacterial suspension to 0.2–0.25. *P. aeruginosa* were then grown at 37°C with shaking at 125 rpm for 70 min. Bacteria were finally washed twice in HEPES/saline (20 mM HEPES, 132 mM NaCl, 5 mM KCl, 1 mM CaCl_2_, 0.7 mM MgCl_2_, 0.8 mM MgSO_4_, pH 7.0) by centrifugation at 1600 × *g* for all *P. aeruginosa* strains for 10 min and finally suspended in H/S for subsequent assays.

To determine the effects of sphingosine on bacterial cell death, we incubated 10,000 CFU of the *P. aeruginosa* strains in 500 µL H/S (pH 7.0) for 45 min with 1 µM, 5 µM, or 10 µM sphingosine or left the samples untreated. We then diluted the samples in H/S, plated aliquots on LB agar plates, and determined the remaining CFU after o/n growth at 37°C.

To determine cardiolipin by the enzymatic assay, 2 × 10^7^ CFU of *P. aeruginosa* were suspended in 500 µL H/S (pH 7.0); sphingosine was added at 1 µM, 5 µM, or 10 µM; and the samples were incubated for 15 or 20 min at 37°C.

To determine cardiolipin by the radioactive labelling assay, 10^5^ CFU of *P. aeruginosa* were suspended in 200 µL H/S (pH 7.0), sphingosine was added, and the samples were incubated for 20 min at 37°C.

Bacteria were complemented with cardiolipin by a 30 min incubation of the bacteria in 10 µM cardiolipin suspended in H/S.

### Measurement of cardiolipin using an enzymatic assay

2 × 10^7^ CFU of the indicated *P. aeruginosa* strains were grown and washed as above, resuspended in H/S, and incubated with 1 µM, 5 µM, or 10 µM sphingosine for 15 or 20 min or left untreated. Samples were washed, resuspended in 130 µL H/S, and lysed three times each 10 sec sonication using a tip sonicator. Cardiolipin was then quantified using a kit from Abcam (#ab241036). To this end, 50 µL aliquots of the lysates were incubated with a probe that specifically reacts with cardiolipin to form a fluorescent dye that can be detected with excitation of 340 nm and emission at 480 nm.

### Measurement of cardiolipin using metabolic labeling

Bacteria were labeled by incubation with 40 µCi [^14^C]acetate [ARC 0173, 58 mCi/mmol] / in 100 mL TSB for 60 min at 37°C. Bacteria were washed once in H/S (pH 7.0), incubated with sphingosine for 20 min in H/S or left untreated, and washed again three times in H/S. Lipids were extracted in 200 µL H_2_O, 10.5 µL 1N HCl, 900 µL CHCl_3_:CH_3_OH (2:1, v/v), 900 µL CHCl_3_, and 150 µL KCl. Samples were vortexed and centrifuged for 5 min at 14,000 rpm, and the organic phase was collected, dried, and dissolved in CHCl_3_:CH_3_OH (1:1, v/v). Samples were then separated by TLC using Silica G60 thin-layer chromatography plates (Merck) using acetic acid, H_2_0, CH_3_OH, and CHCl_3_ (4:8:30:50, v/v/v/v) to separate lipids. Plates were analyzed using a Fuji reader. Cardiolipin was identified by comigration with a standard.

### SDS-mediated lysis of bacteria upon permeabilization of the outer bacterial membrane

*P. aeruginosa* ATCC 27853 or 762 were incubated with 1 µM sphingosine or left untreated for 15 min in 500 µL HEPES/saline (H/S, 132 mM NaCl, 20 mM HEPES [pH 7.0], 5 mM KCl, 1 mM CaCl_2_, 0.7 mM MgCl_2_, 0.8 mM MgSO_4_). After the treatment with sphingosine, 10 mL TSB supplemented with 0.5% sodium dodecyl sulfate and 0.5 mM EDTA were added, the bacteria incubated for 2 hrs at 37°C with shaking at 125 rpm, aliquots of the bacteria were plated on TSA, and CFUs were counted after o/n growth.

### PLA_2_ inhibitors

*P. aeruginosa* strains ATCC 27853 and 762 were grown and labeled with [^14^C]acetate as above, and 2 × 10^8^ CFU/sample were incubated for 10 min with the PLA_2_ inhibitors AACOCF3 (25 µM), VU0364739 (25 µM), OBAA (25 µM), FIPI (50 µM), darapladip (50 µM), or aristolochic acid (5 µM) or the PLC inhibitor U73122 (10 µM), which served as negative control. The given concentrations are final concentration, stocks in DMSO, or 0.9% NaCl were 1,000-fold higher. After the 10 min preincubation period, we added sphingosine at 1 µM, 5 µM, or 10 µM or left the samples untreated. Bacteria were incubated for 15 min, and cardiolipin was determined by extraction of lipids and TLC assays. To determine the survival of the bacteria, we incubated the bacteria with sphingosine for 30 min and then plated aliquots onto TSA plates. Given are the mean ± SD of five independent experiments; ***P <0.001, ANOVA, post hoc Tukey test.

### Statistical analysis and quantification

All data are given as arithmetic means ± SD. Data were analyzed using one-way ANOVA followed by post hoc Tukey tests for all pairwise comparisons of continuous variables from independent groups. All values were tested for normal distribution. We applied Bonferroni correction for multiple testing prior to calculating *P* values for the pairwise comparisons. A *P* value of 0.05 or less was considered indicative of statistical significance. The sample size planning for the continuous variables *in vivo* infection experiments was based on two-sided Wilcoxon-Mann-Whitney tests (free software: G*Power Version 3.1.7 of the University of Duesseldorf, Germany).

## SUPPLEMENTAL MATERIAL

Supplemental material iai.00591-24-s0001.docx Caption for Supplemental movie.

Movie S1 iai.00591-24-s0002.mov TEM tomography of the bacteria treated with 20 μM sphingosine for 10 min indicates how the membrane rolls within short time after treatment with sphingosine, how these rolls are released from the bacteria and that the bacteria finally disintegrate.

ASM does not own the copyrights to Supplemental Material that may be linked to, or accessed through, an article. The authors have granted ASM a non-exclusive, world-wide license to publish the Supplemental Material files. Please contact the corresponding author directly for reuse.

## References

[1] Crouch Brewer S, Wunderink RG, Jones CB, Leeper KV Jr. 1996. Ventilator-associated pneumonia due to Pseudomonas aeruginosa. Chest 109:1019–1029. doi:10.1378/chest.109.4.1019 8635325

[2] McManus AT, Mason AD Jr, McManus WF, Pruitt BA Jr. 1985. Twenty-five year review of Pseudomonas aeruginosa bacteremia in a burn center. Eur J Clin Microbiol 4:219–223. doi:10.1007/BF02013601 3924612

[3] Vidal F, Mensa J, Almela M, Martínez JA, Marco F, Casals C, Gatell JM, Soriano E, Jimenez de Anta MT. 1996. Epidemiology and outcome of Pseudomonas aeruginosa bacteremia, with special emphasis on the influence of antibiotic treatment. Analysis of 189 episodes. Arch Intern Med 156:2121–2126. doi:10.1001/archinte.1996.00440170139015 8862105

[4] Morrison AJ Jr, Wenzel RP. 1984. Epidemiology of infections due to Pseudomonas aeruginosa. Rev Infect Dis 6:S627–S642. doi:10.1093/clinids/6.Supplement_3.S627 6443765

[5] Currie AJ, Speert DP, Davidson DJ. 2003. Pseudomonas aeruginosa: role in the pathogenesis of the CF lung lesion. Semin Respir Crit Care Med 24:671–680. doi:10.1055/s-2004-815663 16088583

[6] Poch DS, Ost DE. 2009. What are the important risk factors for healthcare-associated pneumonia? Semin Respir Crit Care Med 30:26–35. doi:10.1055/s-0028-1119806 19199184

[7] American Thoracic Society. 2005. Infections diseases society of America. Am J Respir Crit Care Med 171:388–416. doi:10.1164/rccm.200405-644ST 15699079

[8] Elborn JS. 2016. Cystic fibrosis. Lancet 388:2519–2531. doi:10.1016/S0140-6736(16)00576-6 27140670

[9] Bhagirath AY, Li Y, Somayajula D, Dadashi M, Badr S, Duan K. 2016. Cystic fibrosis lung environment and Pseudomonas aeruginosa infection. BMC Pulm Med 16:174. doi:10.1186/s12890-016-0339-5 27919253 PMC5139081

[10] Schaffer K. 2015. Epidemiology of infection and current guidelines for infection prevention in cystic fibrosis patients. J Hosp Infect 89:309–313. doi:10.1016/j.jhin.2015.02.005 25791927

[11] Pewzner-Jung Y, Tavakoli Tabazavareh S, Grassmé H, Becker KA, Japtok L, Steinmann J, Joseph T, Lang S, Tuemmler B, Schuchman EH, Lentsch AB, Kleuser B, Edwards MJ, Futerman AH, Gulbins E. 2014. Sphingoid long chain bases prevent lung infection by Pseudomonas aeruginosa*.* EMBO Mol Med 6:1205–1214. doi:10.15252/emmm.201404075 25085879 PMC4197866

[12] Grassmé H, Henry B, Ziobro R, Becker KA, Riethmüller J, Gardner A, Seitz AP, Steinmann J, Lang S, Ward C, Schuchman EH, Caldwell CC, Kamler M, Edwards MJ, Brodlie M, Gulbins E. 2017. β1-integrin accumulates in cystic fibrosis luminal airway epithelial membranes and decreases sphingosine, promoting bacterial infections. Cell Host Microbe 21:707–718. doi:10.1016/j.chom.2017.05.001 28552668 PMC5475347

[13] Bibel DJ, Aly R, Shinefield HR. 1992. Antimicrobial activity of sphingosines. J Invest Dermatol 98:269–273. doi:10.1111/1523-1747.ep12497842 1545135

[14] Fischer CL, Walters KS, Drake DR, Blanchette DR, Dawson DV, Brogden KA, Wertz PW. 2013. Sphingoid bases are taken up by Escherichia coli and Staphylococcus aureus and induce ultrastructural damage. Skin Pharmacol Physiol 26:36–44. doi:10.1159/000343175 23128426 PMC3634627

[15] Azuma MM, Balani P, Boisvert H, Gil M, Egashira K, Yamaguchi T, Hasturk H, Duncan M, Kawai T, Movila A. 2018. Endogenous acid ceramidase protects epithelial cells from Porphyromonas gingivalis-induced inflammation in vitro. Biochem Biophys Res Commun 495:2383–2389. doi:10.1016/j.bbrc.2017.12.137 29278706 PMC5765770

[16] Becam J, Walter T, Burgert A, Schlegel J, Sauer M, Seibel J, Schubert-Unkmeir A. 2017. Antibacterial activity of ceramide and ceramide analogs against pathogenic Neisseria. Sci Rep 7:17627. doi:10.1038/s41598-017-18071-w 29247204 PMC5732201

[17] Martin GE, Boudreau RM, Couch C, Becker KA, Edwards MJ, Caldwell CC, Gulbins E, Seitz A. 2017. Sphingosine’s role in epithelial host defense: a natural antimicrobial and novel therapeutic. Biochimie 141:91–96. doi:10.1016/j.biochi.2017.03.014 28341550

[18] Carstens H, Schumacher F, Keitsch S, Kramer M, Kühn C, Sehl C, Soddemann M, Wilker B, Herrmann D, Swaidan A, Kleuser B, Verhaegh R, Hilken G, Edwards MJ, Dubicanac M, Carpinteiro A, Wissmann A, Becker KA, Kamler M, Gulbins E. 2019. Clinical development of sphingosine as anti-bacterial drug: inhalation of sphingosine in mini pigs has no adverse side effects. Cell Physiol Biochem 53:1015–1028. doi:10.33594/000000194 31854953

[19] Carstens H, Kalka K, Verhaegh R, Schumacher F, Soddemann M, Wilker B, Keitsch S, Sehl C, Kleuser B, Hübler M, Rauen U, Becker AK, Koch A, Gulbins E, Kamler M. 2022. Antimicrobial effects of inhaled sphingosine against Pseudomonas aeruginosa in isolated ventilated and perfused pig lungs. PLoS ONE 17:e0271620. doi:10.1371/journal.pone.0271620 35862397 PMC9302828

[20] Liu Y, Wu Y, Leukers L, Schimank K, Wilker J, Wissmann A, Rauen U, Pizanis N, Taube C, Koch A, Gulbins E, Kamler M. 2024. Treatment of Staphylococcus aureus infection with sphingosine in ex vivo perfused and ventilated lungs. J Heart Lung Transplant 43:100–110. doi:10.1016/j.healun.2023.08.021 37673383

[21] Guest RL, Lee MJ, Wang W, Silhavy TJ. 2023. A periplasmic phospholipase that maintains outer membrane lipid asymmetry in Pseudomonas aeruginosa. Proc Natl Acad Sci U S A 120:e2302546120. doi:10.1073/pnas.2302546120 37463202 PMC10374164

[22] Malinverni JC, Silhavy TJ. 2009. An ABC transport system that maintains lipid asymmetry in the Gram-negative outer membrane. Proc Natl Acad Sci USA 106:8009–8014. doi:10.1073/pnas.0903229106 19383799 PMC2683108

[23] Verhaegh R, Becker KA, Edwards MJ, Gulbins E. 2020. Sphingosine kills bacteria by binding to cardiolipin. J Biol Chem 295:7686–7696. doi:10.1074/jbc.RA119.012325 32327486 PMC7261797

[24] Helander IM, Alakomi H-L, Latva-Kala K, Koski P. 1997. Polyethyleneimine is an effective permeabilizer of Gram-negative bacteria. Microbiology (Reading, Engl) 143:3193–3199. doi:10.1099/00221287-143-10-3193 9353921

[25] Berg Klenow M, Camillus Jeppesen J, Simonsen AC. 2020. Membrane rolling induced by bacterial toxins. Soft Matter 16:1614–1626. doi:10.1039/c9sm01913h 31957755

[26] Hostetler KY, van den Bosch H. 1972. Subcellular and submitochondrial localization of the biosynthesis of cardiolipin and related phospholipids in rat liver. Biochim Biophys Acta 260:380–386. doi:10.1016/0005-2760(72)90052-5 5038258

[27] Tavakoli Tabazavareh S, Seitz A, Jernigan P, Sehl C, Keitsch S, Lang S, Kahl BC, Edwards M, Grassmé H, Gulbins E, Becker KA. 2016. Lack of sphingosine causes susceptibility to pulmonary Staphylococcus aureus infections in cystic fibrosis. Cell Physiol Biochem 38:2094–2102. doi:10.1159/000445567 27184795

[28] Strub GM, Paillard M, Liang J, Gomez L, Allegood JC, Hait NC, Maceyka M, Price MM, Chen Q, Simpson DC, Kordula T, Milstien S, Lesnefsky EJ, Spiegel S. 2011. Sphingosine‐1‐phosphate produced by sphingosine kinase 2 in mitochondria interacts with prohibitin 2 to regulate complex IV assembly and respiration. FASEB J 25:600–612. doi:10.1096/fj.10-167502 20959514 PMC3023391

[29] Feng S, Harayama T, Montessuit S, David FP, Winssinger N, Martinou JC, Riezman H. 2018. Mitochondria-specific photoactivation to monitor local sphingosine metabolism and function. Elife 7:e34555. doi:10.7554/eLife.34555 29376826 PMC5819948

[30] LaBauve AE, Wargo MJ. 2014. Detection of host-derived sphingosine by Pseudomonas aeruginosa is important for survival in the murine lung. PLoS Pathog 10:e1003889. doi:10.1371/journal.ppat.1003889 24465209 PMC3900636

[31] Mackinder JR, Eckstrom K, Wargo MJ, Fisher K, Schutz K, Hinkel LA. 2024. Detection of host-derived sphingosine by Pseudomonas aeruginosa is important for survival in the murine lung. J Bacteriol 206:e0038223. doi:10.1128/jb.00382-23 38411048 PMC10955842

[32] DiGianivittorio P, Hinkel LA, Mackinder JR, Schutz K, Klein EA, Wargo MJ. 2025. The Pseudomonas aeruginosa sphBC genes are important for growth in the presence of sphingosine by promoting sphingosine metabolism. Microbiology (Reading, Engl) 171. doi:10.1099/mic.0.001520 PMC11893366 39791474

[33] Belosludtsev KN, Belosludtseva NV, Kondratyev MS, Agafonov AV, Purtov YA. 2014. Interaction of phospholipase A of the E. coli outer membrane with the inhibitors of eucaryotic phospholipases A₂ and their effect on the Ca²⁺-induced permeabilization of the bacterial membrane. J Membr Biol 247:281–288. doi:10.1007/s00232-014-9633-4 24477786

[34] Burke JE, Dennis EA. 2009. Phospholipase A_2_ structure/function, mechanism, and signaling. J Lipid Res 50 Suppl:S237–S242. doi:10.1194/jlr.R800033-JLR200 19011112 PMC2674709

[35] Dong H, Zhang Z, Tang X, Huang S, Li H, Peng B, Dong C. 2016. Structural insights into cardiolipin transfer from the inner membrane to the outer membrane by PbgA in Gram-negative bacteria. Sci Rep 6:30815. doi:10.1038/srep30815 27487745 PMC4973235

[36] Held K, Ramage E, Jacobs M, Gallagher L, Manoil C. 2012. Sequence-verified two-allele transposon mutant library for Pseudomonas aeruginosa PAO1. J Bacteriol 194:6387–6389. doi:10.1128/JB.01479-12 22984262 PMC3497512

[37] Grassmé H, Jendrossek V, Riehle A, von Kürthy G, Berger J, Schwarz H, Weller M, Kolesnick R, Gulbins E. 2003. Host defense against Pseudomonas aeruginosa requires ceramide-rich membrane rafts. Nat Med 9:322–330. doi:10.1038/nm823 12563314

[38] Mastronarde DN. 2003. SerialEM: a program for automated tilt series acquisition on tecnai microscopes using prediction of specimen position. Microsc Microanal 9:1182–1183. doi:10.1017/S1431927603445911

[39] Schindelin J, Arganda-Carreras I, Frise E, Kaynig V, Longair M, Pietzsch T, Preibisch S, Rueden C, Saalfeld S, Schmid B, Tinevez JY, White DJ, Hartenstein V, Eliceiri K, Tomancak P, Cardona A. 2012. Fiji: an open-source platform for biological-image analysis. Nat Methods 9:676–682. doi:10.1038/nmeth.2019 22743772 PMC3855844

[40] Kremer JR, Mastronarde DN, McIntosh JR. 1996. Computer visualization of three-dimensional image data using IMOD. J Struct Biol 116:71–76. doi:10.1006/jsbi.1996.0013 8742726

