# Supplementary material for: Sphingosine-mediated death of Pseudomonas aeruginosa involves degradation of cardiolipin by the maintenance of outer lipid asymmetry system
Source: Infect Immun. 2025 Mar 10;93(4):e00591-24. doi: 10.1128/iai.00591-24 (PMC11977310; doi:10.1128/iai.00591-24)
Supplement: Supplemental material — Caption for Supplemental movie. [file iai.00591-24-s0001.docx]

Supplementary Figure 1

TEM tomography of the bacteria treated with 20 μM sphingosine for 10 min indicates how the membrane rolls within short time after treatment with sphingosine, how these rolls are released from the bacteria and that the bacteria finally disintegrate. The tomography also shows the formation of novel intracellular membranes.
